# Janus kinase inhibitor overcomes resistance to immune checkpoint inhibitor treatment in peritoneal dissemination of gastric cancer in C57BL/6 J mice

**DOI:** 10.1007/s10120-024-01514-5

**Published:** 2024-05-28

**Authors:** Wan-Ying Du, Hiroki Masuda, Koji Nagaoka, Tomohiko Yasuda, Komei Kuge, Yasuyuki Seto, Kazuhiro Kakimi, Sachiyo Nomura

**Affiliations:** 1 Department of Gastrointestinal Surgery, Graduate School of Medicine, The University of Tokyo, 7-3-1 Hongo, Bunkyo-Ku, Tokyo, 113-8655 Japan; 2 Department of Gastrointestinal Surgery, Nippon Medical School, Tokyo, Japan; 3 Department of Immunotherapeutics, The University of Tokyo Hospital, Tokyo, Japan; 4 Department of Clinical Pharmaceutical Sciences, School of Pharmacy and Pharmaceutical Sciences, Hoshi University, 2-4-41 Ebara, Shinagawa-Ku, Tokyo, 142-8501 Japan

**Keywords:** JAK pathway, Immune checkpoint blocker, Peritoneal metastasis, Immunotherapy

## Abstract

**Background:**

Cancer immunotherapy aims to unleash the immune system’s potential against cancer cells, providing sustained relief for tumors responsive to immune checkpoint inhibitors (ICIs). While promising in gastric cancer (GC) trials, the efficacy of ICIs diminishes in the context of peritoneal dissemination. Our objective is to identify strategies to enhance the impact of ICI treatment specifically for cases involving peritoneal dissemination in GC.

**Methods:**

The therapeutic efficacy of anti-PD1, CTLA4 treatment alone, or in combination was assessed using the YTN16 peritoneal dissemination tumor model. Peritoneum and peritoneal exudate cells were collected for subsequent analysis. Immunohistochemical staining, flow cytometry, and bulk RNA-sequence analyses were conducted to evaluate the tumor microenvironment (TME). A Janus kinase inhibitor (JAKi) was introduced based on the pathway analysis results.

**Results:**

Anti-PD1 and anti-CTLA4 combination treatment (dual ICI treatment) demonstrated therapeutic efficacy in certain mice, primarily mediated by CD8 + T cells. However, in mice resistant to dual ICI treatment, even with CD8 + T cell infiltration, most of the T cells exhibited an exhaustion phenotype. Notably, resistant tumors displayed abnormal activation of the Janus Kinase-Signal Transducer and Activator of Transcription (JAK-STAT) pathway compared to the untreated group, with observed infiltration of macrophages, neutrophils, and Tregs in the TME. The concurrent administration of JAKi rescued CD8 + T cells function and reshaped the immunosuppressive TME, resulting in enhanced efficacy of the dual ICI treatment.

**Conclusion:**

Dual ICI treatment exerts its anti-tumor effects by increasing tumor-specific CD8 + T cell infiltration, and the addition of JAKi further improves ICI resistance by reshaping the immunosuppressive TME.

**Supplementary Information:**

The online version contains supplementary material available at 10.1007/s10120-024-01514-5.

## Introduction

The incidence and mortality rates of gastric cancer (GC) remain high, despite significant advancements in cancer treatment. According to GLOBOCAN 2020 data, GC ranks fifth in incidence and fourth in mortality globally [1]. The metastatic progression of GC involves three main pathways: lymphatic metastasis, hematogenous metastasis, and peritoneal dissemination. While lymphatic and hematogenous metastases are common dissemination processes in solid cancers, peritoneal dissemination is more prevalent, accounting for approximately 53–60% of GC-related deaths [2, 3]. Patients with peritoneal dissemination of GC face devastating complications, such as bowel obstruction and massive ascites, resulting in a dismal 5-year overall survival rate of only 2%. This includes patients who have only microscopic free cancer cells in peritoneal cavity and no macroscopically visible peritoneal nodules [4, 5]. A retrospective study revealed that roughly 10% of patients who underwent gastrectomy developed peritoneal recurrence. In addition, among stage IV patients, approximately 37.2% experienced peritoneal dissemination over time [6, 7].

Conventional chemotherapy targets cancer cell proliferation but often falls short in providing a durable response to metastatic tumors [8]. In contrast, cancer immunotherapy focuses on harnessing the immune system’s potential to eliminate cancer cells. It is effective for a long time against responsive cancers. T cell proliferation and activation rely not only on the recognition and binding of the T cell receptor to antigens presented by antigen-presenting cells in major histocompatibility complexes (MHCs) but also on the tight regulation of co-stimulatory and inhibitory signaling molecules [8]. Tumors evade immune surveillance by exploiting immune checkpoint pathways.

To address this challenge, monoclonal antibodies (mAbs) have been developed to block immune checkpoint molecules and unleash the anti-tumor immune response. Immune checkpoint inhibitor (ICI), such as those targeting programmed death 1 (PD1) and cytotoxic T-lymphocyte-associated protein 4 (CTLA4), have both demonstrated remarkable anti-tumor effects in patients with advanced melanoma, renal cell carcinoma, non-small cell lung cancer, and malignant pleural mesothelioma [9]. Extensive preclinical and clinical studies are currently underway to further elucidate their efficacy, mechanisms, and strategies for overcoming tumor tolerance [10, 11]. While the Food and Drug Administration (FDA) in the USA and the Ministry of Health, Labour and Welfare in Japan have also approved anti-PD1 antibody in combination with chemotherapy as a first-line treatment for GC patients, its efficacy in those with peritoneal dissemination is not significantly pronounced [9, 12].

In this study, we employed an immunocompetent mouse model of GC peritoneal dissemination, which we developed, to investigate the alterations in the tumor microenvironment (TME) and identify strategies to enhance the effect of ICI treatment for peritoneal dissemination of GC.

## Methods

See supplementary materials for details.

## Results

### The therapy with anti-PD1 and CTLA4 antibodies against GC peritoneal dissemination

**Fig. 1:**
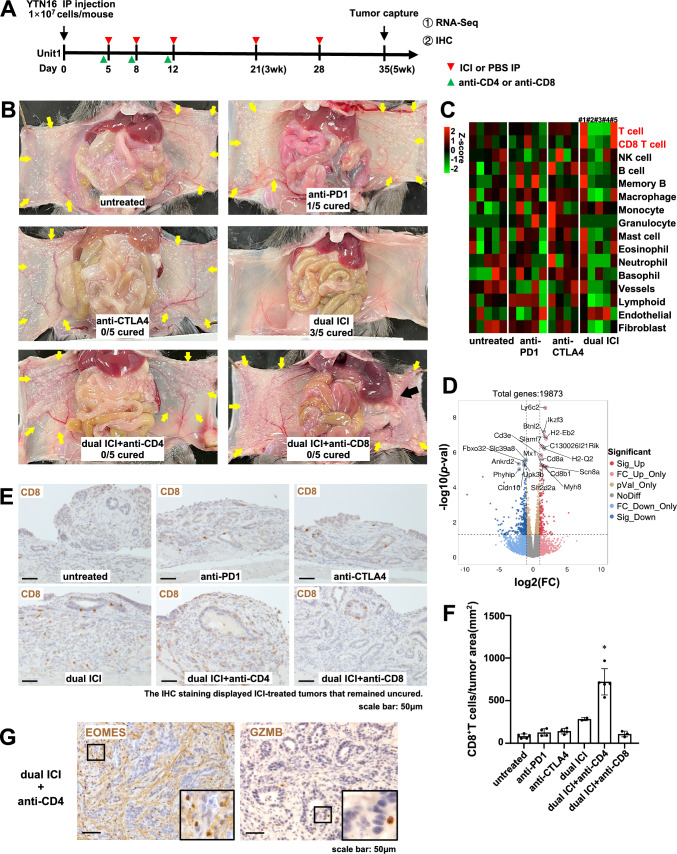
Dual ICI treatment outperformed monotherapy. CD4 + and CD8 + T cells were both indispensable for the anti-tumor effect. **A** Treatment timeline in female C57BL/6 J GC peritoneal dissemination model. **B** Representative macroscopic images of peritoneal dissemination tumor of mice on day 35. Three out of five mice demonstrated improved efficacy with dual ICI treatment compared to the monotherapy group. However, the clearance of CD4 + or CD8 + T cells, in conjunction with dual ICI treatment, resulted in exacerbated peritoneal dissemination of the tumor. Adhesions of the peritoneum and liver could be seen in the CD8 + T cells depletion group (black arrow). **C** Quantification of immune-infiltrating cells based on bulk RNA-seq data using mMCP-counter analysis. The dual ICI group (mice #1 and #5) exhibited significantly higher levels of immune-infiltrating CD8 + T cells compared to other groups. By the fifth week, the peritoneal dissemination in mouse #2, #3, and #4 had been completely cured. Consequently, the observed distinct infiltration pattern in the heatmap suggested a quiescent immune response, indicating a cessation of immune activation. **D** The volcano plot illustrated the DEGs between the dual ICI and untreated groups. The x-axis represented the log2 fold change, indicating the magnitude of gene expression change, while the y-axis represented the statistical significance. Red solid dots highlighted the upregulated DEGs in the dual ICI group, exceeding the significance thresholds (*p* < 0.05 and fold change > 2). **E** Representative images of IHC staining of CD8 + T cells on day 35. **F** Enhanced infiltration of CD8 + T cells was observed in the dual ICI treatment group, particularly with CD4 + T cell depletion, resulting in a significant increase compared to other groups. **G** In the dual ICI + anti-CD4 treatment group, IHC staining revealed that although there was significant CD8 + T cell infiltration, only a small proportion exhibited GZMB + cytotoxic phenotype, while the majority displayed an EOMES + exhausted phenotype. Scale bar: 50 μm; inset: magnified view of the boxed area. *p < 0.05, one-way ANOVA with Tukey's multiple comparisons test

To evaluate the efficacy of ICI in the context of peritoneal dissemination of GC, we established a female C57BL/6 J GC peritoneal dissemination mouse model [13, 14]. The grouping and treatment timeline are depicted (Fig. 1A, supplementary Fig. 1A). Following the implantation of YTN16 cells on day 0, IP administration of ICI or PBS commenced on days 5, 8, 12, 21, and 28. Concurrently, T-cell depletion was achieved via IP injection of anti-CD4 or anti-CD8 mAb on days 4, 7, and 11 for confirming the therapeutic effect originated from T cell. On day 35, samples were collected for analysis. Beginning in the fourth week following YTN16 cell transplantation, mice displayed a declining trend in weight, excluding those in the dual ICI treatment group (supplementary Fig. 1B). One mouse out of 5 mice treated with anti-PD1 monotherapy was cured, but none out of 5 mice treated with anti-CTLA4 monotherapy was cured. In the anti-CTLA4 group, one mouse, which died before the autopsy, was excluded from the analysis (Supplementary Fig. 1C). Remarkably, among the five mice subjected to dual ICI treatment, three exhibited significant improvements in macroscopic disseminated nodules compared to the monotherapy group (Fig. 1B, Supplementary Fig. 1D). The treatment efficiency was abrogated upon introducing CD4 + or CD8 + T cell depletion in conjunction with dual ICI treatment. In the dual ICI + anti-CD8 group, two mice, which died before the autopsy, were excluded from the analysis. In these mice, a notable reversal in therapeutic outcomes was observed, marked by an exacerbation in peritoneal tumor dissemination and even pronounced adhesions and jaundice (Fig. 1B, Supplementary Fig. 1E). Immune infiltrating cells were quantified based on bulk RNA-seq data using mMCP-counter analysis (Fig. 1C). Because peritoneal dissemination had been entirely eradicated in three mice from the dual ICI treatment group by the fifth week, distinct infiltration patterns on the heatmap suggested a state of “post-war” quiescent immune response (Fig. 1C, mice #2, #3, and #4). However, even among the uncured cases, mice from the dual ICI treatment group (Fig. 1C, mice #1, #5) displayed significantly higher levels of immune-infiltrating CD8 + T cells compared to the mice in the other groups. In addition, the volcano plot illustrated the differentially expressed genes (DEGs) between the dual ICI and untreated group, with upregulated DEGs marked with red solid dots. The notable upregulation of gene expression in Cd8a, Cd8b1, H2-Eb2, H2-Q2, and Ly6c2 indicated enhanced immune activation and potential involvement of MHC-related pathways, as well as an association with inflammation-related processes in dual ICI-treated mice (Fig. 1D).

The presence of CD8 + T cell infiltration was verified via immunohistochemical (IHC) staining (Fig. 1E, F). Remarkably, in the uncured mice from the dual ICI treatment group, there was a substantial increase in CD8 + T cell infiltration compared to the monotherapy and untreated groups. Furthermore, an intriguing observation arose when combining dual ICI treatment with CD4 + T cell depletion. Specifically, though substantial CD8 + T cell infiltration was evident, the majority exhibited an EOMES + exhausted phenotype, while only a minority displayed the GZMB + cytotoxic phenotype (Fig. 1G). In these unremitting tumors, a substantial infiltration of CD68 + macrophages, Ly6G + neutrophils, and Foxp3 + regulatory T cells (Tregs) were observed, indicating the formation of an immunosuppressive TME. It is worth noting that, despite a significant reduction in Foxp3 + Tregs infiltration following anti-CD4 and anti-CD8 treatment, the tumor did not show any improvement (supplementary Fig. 2).

### Dual ICI treatment exerted anti-tumor immune effects by enhancing the infiltration of tumor neoantigen-specific CD8 + T cells

**Fig. 2:**
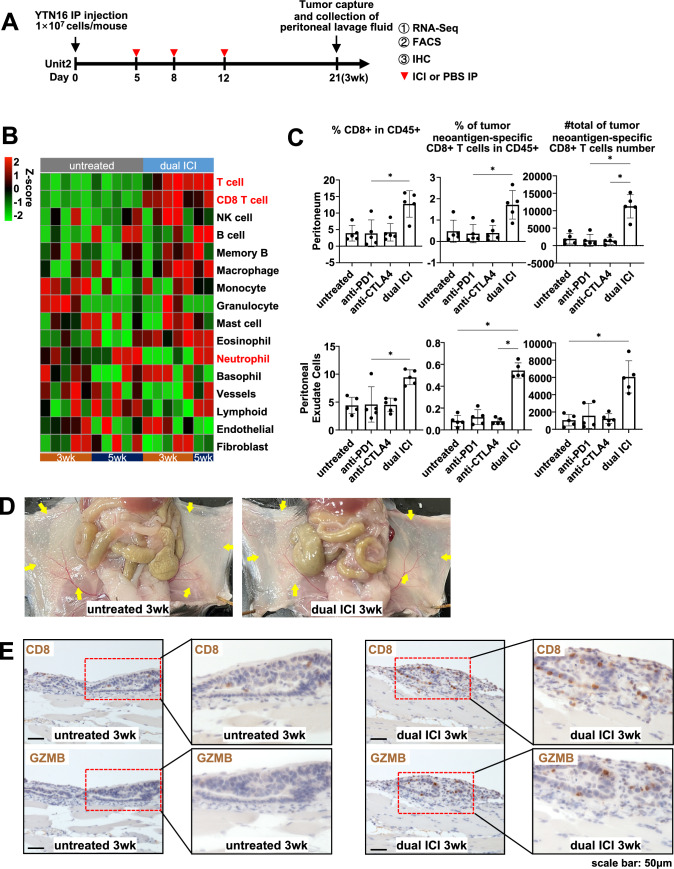
Dual ICI treatment exerted anti-tumor immune effects by enhancing the infiltration of tumor neoantigen-specific CD8 + T cells. **A** Treatment timeline in female C57BL/6 J GC peritoneal dissemination model. **B** The quantification of immune cell infiltration during the anti-tumor immune response was conducted at both the early stage (3wk) and the late stage (5wk, uncured mice) using mMCP-counter analysis. Dual ICI treatment resulted in increased CD8 + T cell infiltration compared to the untreated group at both time points. Notably, at the early time point (3wk), the dual ICI group exhibited relatively fewer neutrophil infiltrations compared to the untreated group. However, in the late stage of dual ICI treatment, increased neutrophil infiltration was observed in uncured tumors. **C** FACS analysis was performed on peritoneal tumor and peritoneal exudate cells at the early time point (3wk). Significant increase observed in tumor neoantigen-specific CD8 + T cells in the dual ICI treatment group. Tumor neoantigen-specific CD8 + T cells were defined as composed of mCdt1-H-2 Kb-dimer + CD8 + T cells, mZfp106-H-2D^b^-dimer + CD8 + T cells, and mScarb2-H-2D^b^-dimer + CD8 + T cells. *p < 0.05, Kruskal–Wallis test with Dunn’s multiple comparison test. **D** Representative images of peritoneal dissemination of mice on day 21. **E** IHC staining of CD8 and GZMB showed enhanced infiltration of GZMB + cytotoxic T cells into the tumor following dual ICI treatment at the early time point (3wk). Scale bar: 50 μm; Inset: a magnified view within the red dashed line, presented in a black frame on the right

To observe treatment affected tumor microenvironment at an earlier time point, before tumor disappearing for the dual ICI therapy, we euthanized the mice at the third week (Fig. 2A). The study involved IP implantation of YTN16 cells on day 0, followed by IP treatments with ICI or PBS on days 5, 8, and 12. After 21 days, mice were euthanized, and peritoneal tumors were collected for RNA-seq, fluorescence activated cell sorting (FACS), and IHC analysis. In addition, peritoneal lavage fluid was examined for FACS analysis of peritoneal exudate cells.

The immune cell composition at the third week was depicted in a pie chart format using CIBERSORT. The dual ICI treatment group exhibited an increased relative proportion of CD8 + T cells, whereas in the untreated group, neutrophils accounted for a higher proportion (supplementary Fig. 3). Similarly, at this earlier time point (3wk), we also conducted mMCP-counter analysis and compared the results with those from the later time point (5wk). Dual ICI treatment resulted in increased CD8 + T cell infiltration compared to the untreated group at both time points. Notably, at the earlier time point (3wk), the dual ICI group exhibited relatively fewer neutrophil infiltrations compared to the untreated group. However, in the late stage of dual ICI treatment (5wk), increased neutrophil infiltration was observed in uncured tumors (Fig. 2B). This shift in neutrophil infiltration within tumors was also confirmed by IHC for ly6G, a neutrophil marker (supplementary Fig. 4A).

Previously, we have identified 3 neo-antigens for YTN16 cells, including mCdt1, mZfp106, and mScarb2 peptides [15]. Flow cytometry analysis revealed increased CD8 + T cell in tumor-bearing peritoneum and peritoneal lavage fluid with dual ICI treatment, along with a significant rise in neo-antigen-specific CD8 + T cells at the earlier time point (Fig. 2C). Notably, there were no significant differences observed in the levels of NK cells, B cells, and CD4 + T cells among groups (Supplementary Fig. 4B).

Representative images showed similar peritoneal tumor sizes among mice in the untreated and dual ICI group (Fig. 2D). IHC analysis revealed an increase in CD8 + T cell infiltration in the tumor area following dual ICI treatment, compared to the untreated group. Remarkably, most of these CD8 + T cells exhibited a GZMB + cytotoxic T cell phenotype, while the limited number of CD8 + T cells present in the untreated group did not show GZMB expression (Fig. 2E).

The above analysis indicated that dual ICI treatment exerted potent anti-tumor effects by enhancing the infiltration of tumor neoantigen-specific CD8 + T cells. Notably, neutrophil infiltration displayed varying patterns, with reduced presence at the early stage and increased infiltration in uncured tumors during late-stage treatment. It is conceivable that during the treatment process of dual ICI, the tumor response gradually shows different trends. Some mice may manifest a sustained response to ICI, resulting in a gradual tumor clearance, whereas others, despite the activation of an immune response, may develop resistance to ICI for unspecified reasons, subsequently leading to further tumor progression.

### Excessive activation of JAK-STAT pathway in dual ICI-resistant tumors

**Fig. 3:**
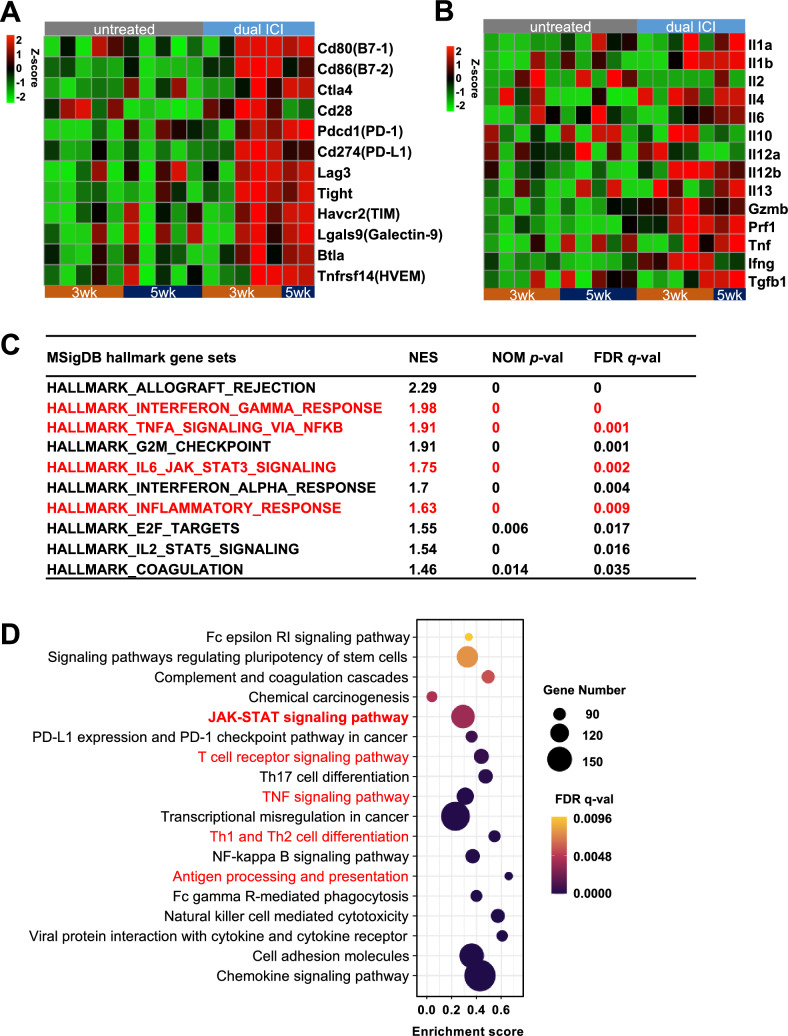
Enhanced activation of the JAK-STAT pathway observed in dual ICI-uncured mice compared to untreated mice. **A**,** B** Heatmap illustrating the expression of immune checkpoint molecules and cytokines, cytotoxic molecules at both early (3wk) and late (5wk) time points in untreated and dual ICI-uncured mice. **C** GSEA of MSigDB hallmark gene sets in dual ICI-uncured mice compared to untreated Mice. The top 10 enriched gene sets in dual ICI-uncured mice were displayed. **D** Enrichment analysis of KEGG pathways was performed using STRING. Pathways enriched in dual ICI-uncured mice were displayed in a bubble chart

To investigate the underlying causes of resistance in dual ICI treatment, we conducted an examination of immune checkpoints, including both inhibitory and co-stimulatory molecules, along with the assessment of relevant cytokine and cytotoxic molecule expression. Notably, following dual ICI treatment, several immunosuppressive checkpoint genes showed significant upregulation, such as Pdcd1, Ctla4, Lag3, Tight, Tim, Lgals9, Btla, and Tnfrsf14. This upregulation was observed in some mice during the early stage and persisted in uncured mice during the late stage following dual ICI treatment. In contrast, immune-positive stimulatory signals such as CD28, were expressed during the early stage (3wk) but markedly declined in the late stage (Fig. 3A). In response to dual ICI treatment, the expression levels of interleukin (IL)-1, 4, 6, and 13 increased indicating an elevated inflammatory response and potential tumor-promoting involvement. Notably, IL-12, crucial for T cell activation and essential for anti-tumor immune responses, increased after dual ICI treatment but declined in late-stage (5wk) ICI-resistant mice. Granzyme B (Gzmb), Perforin-1 (Prf1), Tumor Necrosis Factor (Tnf), and Interferon Gamma (Ifng) also exhibited noticeable upregulation following dual ICI treatment. However, a declining trend in Ifng expression was observed in the late-stage (5wk) ICI-resistant mice, concomitant with an increase in Transforming Growth Factor Beta-1 (Tgfb1) associated with tumor epithelial–mesenchymal transition (EMT) (Fig. 3B). In addition, we generated a heatmap to assess changes in chemokine expression following dual ICI treatment, revealing significant upregulation of multiple chemokines, including Ccl2,17,20,28, and Cxcl 1,2,3,5,11,16. These chemokines, crucial for regulating immune cell chemotaxis and inflammatory processes, highlight the potential impact of dual ICI treatment on modulating the intricate network of chemotactic signaling. Such changes may facilitate the recruitment and activation of immune cell populations, ultimately influencing the immune landscape within the TME (Supplementary Fig. 5).

We employed gene set enrichment analysis (GSEA) to analyze the top ten enriched Molecular Signatures Database (MSigDB) hallmark gene sets with corresponding enrichment scores in dual ICI-uncured group compared to the untreated group (Fig. 3C). Following the dual ICI treatment, pathways related to immune response, such as INTERFERON_GAMMA_RESPONSE and TNFA_SIGNALING, showed discernible upregulation. Particularly noteworthy was the activation of the IL6_JAK_STAT3_SIGNALING pathway, a key driver of pro-inflammatory signaling. This pathway plays a pivotal role in diverse cellular processes such as cell proliferation, differentiation, and immune regulation. Furthermore, the INFLAMMATORY_RESPONSE pathway also showed substantial activation in the dual ICI-uncured group, further highlighting the complex interplay of inflammatory mechanisms (Supplementary Fig. 6A).

Enrichment analysis of Kyoto Encyclopedia of Genes and Genomes (KEGG) provided an encompassing perspective on the molecular processes influenced by the dual ICI treatment (Fig. 3D). The activation of the antigen processing and presentation pathway, T cell receptor signaling pathway and Th1 and Th2 cell differentiation pathways indicated the potential enhancement of antigen recognition by immune cells, heightened T cell activation and differentiation, which were critical for an effective immune response against tumors. Notably, KEGG analysis also revealed the activation of the JAK-STAT pathway.

The Gene Ontology (GO) enrichment analysis yielded a wide spectrum of enriched terms across Biological Process (BP), Cellular Component (CC), and Molecular Function (MF) categories in dual ICI-uncured mice compared to untreated mice (Supplementary Fig. 6B). BP terms included ‘Positive regulation of T cell activation’ and ‘Myeloid leukocyte activation’ suggested associations with immune modulation and regulation. The enriched CC terms, such as ‘Kinetochore’ and ‘Immunological synapse’, offered insights into potential cellular compartments and structures influenced by the therapeutic interventions. Within the MF category, terms like ‘Cytokine receptor activity’ and ‘Phosphoprotein binding’ implied involvement in signaling and interaction mechanisms. These findings collectively provided clues about how interventions may impact various immunological and molecular processes, contributing to our understanding of the complex interplay between immune responses and TME dynamics in the context of GC peritoneal dissemination.

### JAK-STAT pathway was considered a potential therapeutic target

**Fig. 4:**
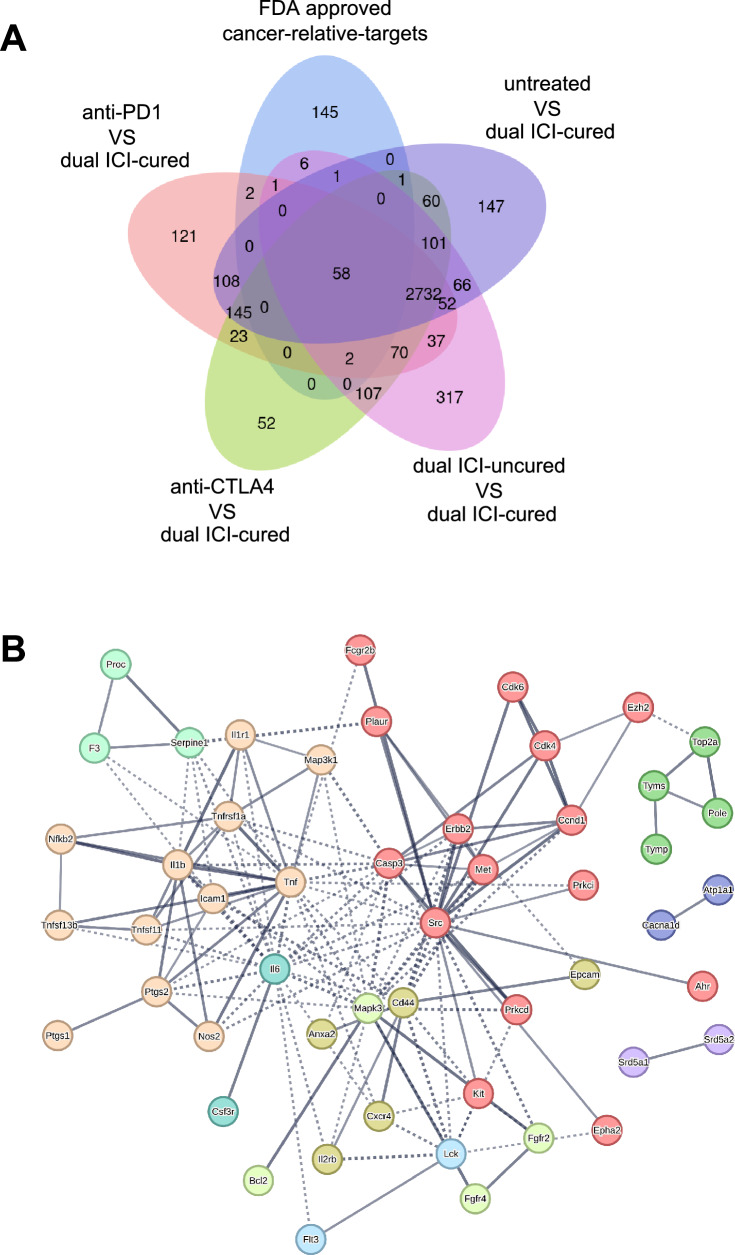
The JAK-STAT pathway was considered a potential therapeutic target.** A** A Venn diagram that is generated by overlapping the DEGs (from each group compared to the dual ICI-cured group) with the FDA-approved cancer-related targets. **B** A PPI network diagram was constructed using the overlapped 58 target proteins. The network was filtered to retain high-confidence interactions (0.7) and visualize protein interaction clusters using MCL clustering while hiding disconnected nodes

We compared the gene profiles of the anti-PD1, anti-CTLA4 monotherapy group, the dual ICI-uncured group, and the untreated group with those of the dual ICI-cured mice. We screened for DEGs from each group and identified potential therapeutic targets by overlapping them with FDA-approved 216 cancer relative targets (obtained from Human Protein Atlas). This analysis was visually represented in a Venn diagram, illustrating a total of 58 potential therapeutic targets crucial to understanding the tumor’s progression (Fig. 4A). A protein–protein interaction (PPI) network diagram was constructed using these 58 target proteins. The network was filtered to retain only high-confidence interactions (0.7), while hiding disconnected nodes. In addition, Markov Cluster Algorithm (MCL) clustering was utilized to visualize the protein interaction clusters (Fig. 4B). Local network clustering analysis using the String platform revealed enrichments in key biological terms, such as ‘apoptosis,’ ‘NF-kappa B signaling,’ ‘TNF receptor superfamily,’ ‘JAK-STAT pathway,’ and ‘vascular endothelial growth factor signaling pathway.’ These findings shed light on potential therapeutic avenues (Supplementary Table 2). Consequently, whether it relates to the tumor’s initiation and progression or its resistance to ICI treatment, the activity of the JAK-STAT pathway assumes a profoundly pivotal role. This exploration suggested a promising therapeutic potential of JAK inhibitor.

### Ruxolitinib (JAK1/2 inhibitor) synergistically improved the immunosuppressive TME in mice with dual ICI resistance

On the foundation of dual ICI application, we administered oral treatment with a JAK inhibitor Ruxolitinib in an attempt to ameliorate resistance to ICI treatment by mitigating excessive inflammatory responses following ICI-induced immune activation. Female C57BL/6 J mice, aged 5 weeks, were divided into four groups: untreated, JAKi, dual ICI, and dual ICI + JAKi.

**Fig. 5:**
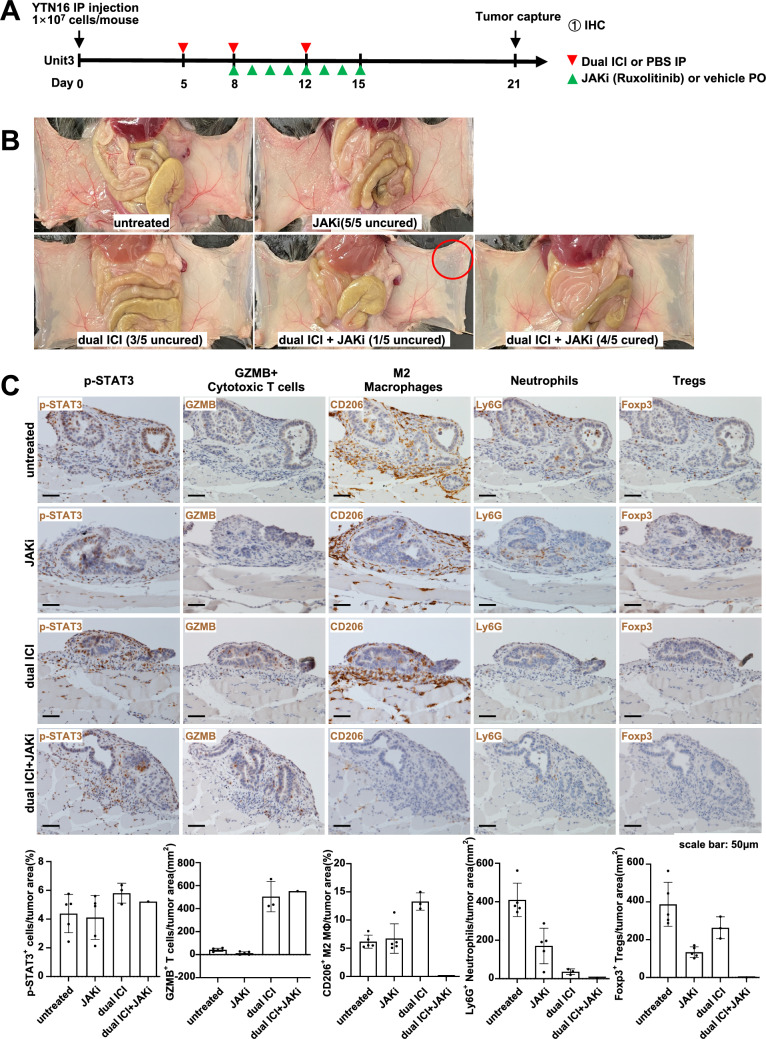
Ruxolitinib in combination with dual ICI treatment further enhanced the anti-tumor effect. **A** Treatment timeline in female C57BL/6 J GC peritoneal dissemination model. **B** Representative macroscopic images of peritoneal dissemination of mice on day 21. Red circle indicated the residual tumor site in the only mouse from the dual ICI + JAKi group. **C** Representative images of IHC staining of p-STAT3, cytotoxic T cells, M2 macrophages, neutrophils, and Tregs on day 21. The dual ICI + JAKi group displays the only uncured mouse with a local residual tumor in the peritoneum. The IHC staining of this mouse was characterized by an abundance of GZMB + cytotoxic T cell infiltration, accompanied by minimal presence of M2 macrophages, neutrophils and Tregs. Scale bar: 50 μm

Dual ICI treatment was initiated on days 5, 8, and 12 following YTN16 implantation. Starting from day 8, Ruxolitinib was administered orally at a daily dose of 30 mg/kg for 1 week. The mice were euthanized on day 21 (Fig. 5A). In the untreated and JAKi monotherapy groups, no improvement was observed, and in the dual ICI group, 3 out of 5 mice exhibited resistance. However, to our surprise, the group that received the combination therapy of dual ICI and JAKi achieved complete tumor clearance in 4 out of 5 mice, with only one mouse showing residual tumor in a small area (Fig. 5B red circle). After subsequent IHC staining, an abundance of GZMB + cytotoxic T cells was discovered within the tumor of this mouse, resembling that observed in the dual ICI group. Furthermore, minimal presence of M2 macrophages, neutrophils, and Tregs was noted. In addition, by day 21, following cessation of JAKi administration on day 15, the expression of p-STAT3 was similar across all groups (Fig. 5C).

**Fig. 6:**
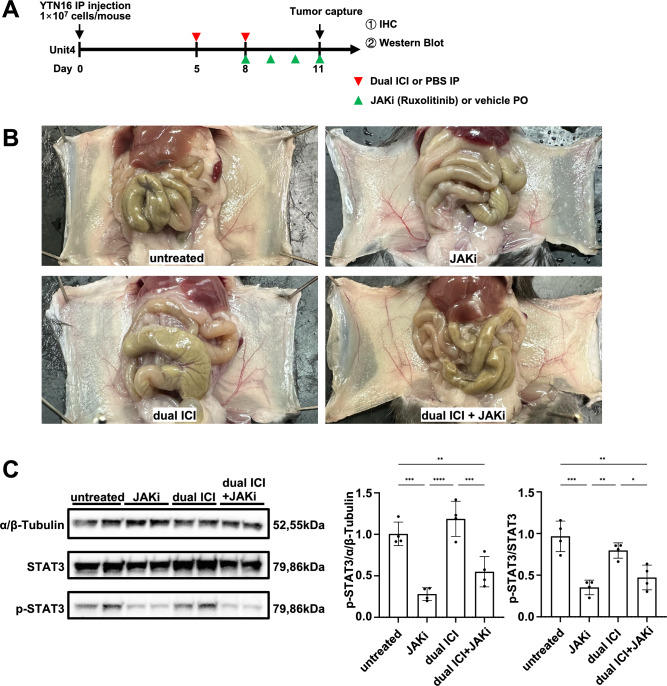
Ruxolitinib inhibited the activation of the JAK-STAT3 pathway in both JAKi and dual ICI + JAKi group. **A** Treatment timeline in female C57BL/6 J GC peritoneal dissemination model. **B** Representative macroscopic images of peritoneal dissemination of mice on day 11. The extent of peritoneal dissemination was consistent across all groups. **C** Western blot analysis and quantification of p-STAT3 protein bands following JAKi administration. *p < 0.05, **p < 0.01, ***p < 0.001, ****p < 0.0001, one-way ANOVA with Tukey’s multiple comparisons test

**Fig. 7:**
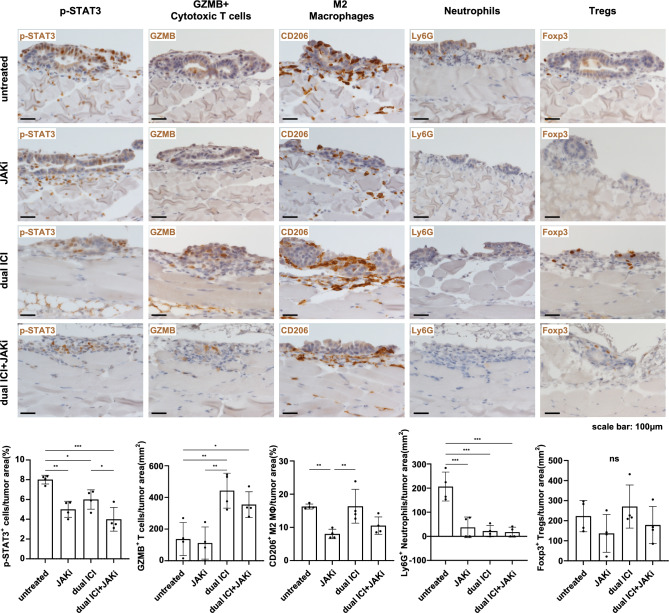
Dual ICI in combination with JAKi reshaped the immunosuppressive microenvironment. Representative images of IHC staining of p-STAT3, cytotoxic T cells, M2 macrophages, neutrophils, and Tregs on day 11. Scale bar: 100 μm; *p < 0.05, **p < 0.01, ***p < 0.001, one-way ANOVA with Tukey’s multiple comparisons test. *ns* not significant, Kruskal–Wallis test with Dunn’s multiple comparison

To collect tumor tissue for analysis before complete clearance, we modified the treatment schedule. We administered dual ICI treatment on day 5, followed by a second dose on day 8. JAKi was administered from day 8 to 11, and the mice were euthanized 3 h after the last dose of JAKi on day 11 (Fig. 6A). Upon dissection, it was observed that the extent of peritoneal dissemination was consistent across all groups (Fig. 6B). Building on this observation, we conducted Western blot analysis to assess the impact of the JAK inhibitor on p-STAT3 protein expression. Following JAKi administration, we observed a significant decrease in the intensity of the p-STAT3 protein bands in comparison to the untreated group (Fig. 6C), suggesting effective suppression of JAK-STAT3 pathway activation. In addition, the IHC results for p-STAT3 suggested its suppression by JAKi (Fig. 7). The IHC results showed that in the treatment regimens involving dual ICI, there was a significant infiltration of GZMB + cytotoxic T cells, while in the regimens involving JAKi, the expression of CD206 + M2-type macrophages was relatively lower. Ly6G + neutrophils in groups that received either JAKi, the dual ICI treatment regimen, or a combination of both did not show a significant increase, unlike the untreated group. While dual ICI treatment showed a modest increase in Foxp3 + Tregs, although it did not reach statistical significance. The therapeutic effectiveness of dual ICI + JAKi was accompanied by both GZMB + cytotoxic T cell infiltration and the lower Ly6G + neutrophil infiltration.

## Discussion

While ICIs have demonstrated promising results in some clinical trials for GC [9, 16], it is crucial to underscore that their effectiveness is less favorable when GC is accompanied by peritoneal dissemination [12]. Our research indicated that dual ICI treatment, combining anti-PD1 and anti-CTLA4, demonstrated superior treatment outcomes in peritoneal dissemination of GC compared to monotherapy. Nevertheless, approximately half of the mice still exhibited resistance to dual ICI, highlighting that, despite a substantial CD8 + T cell infiltration induced by dual ICI treatment, some tumors remained resistant. Furthermore, we found that dual ICI treatment also led to the overexpression of multiple immune checkpoint molecules, gradually transforming cytotoxic T cells into exhausted T cells. This concurrent formation of an immunosuppressive TME, a critical mechanism used by tumors to evade the immune system, significantly affected the efficacy of dual ICI. With an increasing number of therapeutic drugs targeting immune checkpoints being developed, the concurrent rise in side effects and the significant financial burden associated with combination therapies create challenges and complexities in the selection of treatment approaches. Similarly, therapeutic strategies targeting neutrophils, macrophages, or Tregs have also demonstrated potential effectiveness. Nevertheless, it is essential to acknowledge the intricate interplay and regulation among various cell types within the immunosuppressive TME, which poses a complex challenge [17, 18]. Anti-CTLA4 treatment was initially postulated to rejuvenate dysfunctional T cells. However, in mouse models, its anti-tumor effects have been shown to depend on the depletion of Tregs within the TME through Fcγ receptor-mediated antibody-dependent cellular cytotoxicity (ADCC) [19]. Nonetheless, there have been reports of a lack of depletion of FOXP3 + Tregs in human tumors [20].

In Nagaoka et al.’s research based on the YTN16 subcutaneous tumor model, anti-CTLA4 mAb monotherapy effectively eradicated more than 80% of the tumors. Moreover, this robust anti-tumor effect was nullified when anti-CD8 mAb was concurrently administered [15, 21]. In our study, the use of anti-CTLA4 monotherapy did not achieve the same efficacy, and there was no notable reduction in Tregs, which may be related to the specificity of the TME in peritoneum dissemination model and the lack of substantial changes in NK cells following ICI treatment. In fact, our study found that the strong anti-tumor effect of ICI, which relies on CD8 + T cells, was canceled after the application of anti-CD8 antibodies. Ueha S. et al. reported that in subcutaneous tumor models (B16F10, Colon 26, or Lewis lung carcinoma), treatment with the anti-CD4 mAb alone demonstrated robust anti-tumor effects that exceeded those elicited by CD25 + Treg depletion or other ICI monotherapies. Combining the anti-CD4 mAb with anti-PD1 or anti-PDL1 mAbs significantly enhanced treatment efficacy [22]. While in our YTN16 peritoneal dissemination model, the combination of anti-CD4 mAb with dual ICI effectively depleted Tregs, leading to a substantial influx of CD8 + T cells into the tumor, the tumor still showed no improvement. Single-cell level studies have revealed the existence of multiple Treg subtypes, each fulfilling intricate roles within the TME. Focusing solely on targeting a specific cell type may not yield the anticipated outcomes [23, 24]. Therefore, further investigation into the mechanisms governing the interactions among these cells is imperative for a comprehensive understanding.

We initially focused on the JAK-STAT pathway due to its consistent presence in all enrichment pathways based on the RNA-seq results we conducted. The activation of the JAK-STAT signaling pathway is believed to promote the development and progression of various inflammatory diseases, hematologic malignancies, solid tumors, and autoimmune disorders. It holds a pivotal position in activating transcriptional programs in response to a variety of soluble mediators including cytokines, growth factors, IFNs, ILs, and colony-stimulating factors [25]. The JAK1/2-selective inhibitor Ruxolitinib has received FDA approval for treating myelofibrosis, polycythemia vera, graft versus host disease, atopic dermatitis, and vitiligo. It has demonstrated the ability to reduce STAT3 activation in preclinical models of several solid tumors [26–28]. IFN signaling through the JAK-STAT family is well known for its roles in immune stimulation and promoting anti-tumor immunity. However, the dual nature of IFN’s functions manifests over time, especially in the context of chronic inflammation. Prolonged IFN signaling, coupled with persistent antigen exposure, can lead to chronic inflammation and promote inflammation-mediated tumor development. This highlights the potential immunosuppressive functions of IFNs, which govern a complex resistance program to ICI treatment [29–31]. In the early stage of GC peritoneal dissemination mouse models treated with dual ICI, the significant increase in IFN-gamma expression exerted potent anti-tumor effects. However, as IFN signaling persisted, the gradual expression of multiple inhibitory immune checkpoints occurred, leading to the transformation of cytotoxic T cells into exhausted T cells. Furthermore, we observed significant alterations in multiple factors following dual ICI treatment. Key factors such as IL-1β, IL-6, and IL-13 emerged as pivotal players in TME inflammation activation. Moreover, the dynamics of chemokine receptors, such as CCR2, CCR5, and CXCR2, are crucial in coordinating the recruitment and modulation of various immune cell subsets, including neutrophils, macrophages, and Tregs. The intricate interplay fosters the development of an immunosuppressive TME.

The concurrent activation of anti-tumor immune signaling pathways and the JAK-STAT3 pathway suggested a complex regulatory interplay that contributed to tumor resistance against ICI treatment. It is known that IL-6-induced STAT3 activation promotes cell survival, tumor EMT in vitro [32, 33], and is associated with elevated p-STAT3 levels in GC tissues [34]. In Zak J. et al.’s study on JAK1-/- and JAK2-/- MC38 tumor-bearing B6 mice, monocytes showed significantly higher levels of MHC-II transcript and protein in tumors treated with dual ICI + JAKi compared to those treated with dual ICI alone. These results suggested that while JAK1/2 deficiency altered the tumor-immune properties of syngeneic tumors, Ruxolitinib’s immunomodulatory effect was at least partially independent of tumor cell-intrinsic JAK signaling [35]. While inhibiting the excessive activation of the JAK-STAT pathway, whether directly hindering tumor proliferation or modulating tumor immunity, can offer therapeutic benefits, the broader utilization of JAK inhibitors in cancer treatment has faced challenges due to their immunosuppressive characteristics [36]. Given the crucial involvement of JAK-STAT signaling in antigen presentation and T cell recruitment, completely inhibiting JAK1/2 activity is unlikely to enhance anti-tumor immunity or the response to ICI treatment. Therefore, optimal dosing of JAK inhibitors is likely pivotal for their success in cancer immunotherapy [37, 38]. Our results suggested that 30 mg/kg of JAKi administration, instead of suppressing crucial anti-tumor immunity, synergized with dual ICI treatment to reshape the immunosuppressive TME by attenuating excessive inflammatory response and reducing infiltration of tumor-associated neutrophils and macrophages. Mathew, et al. reported that an early response to anti-PD1 treatment was associated with a low baseline level of inflammation, followed by the induction of high and persistent inflammatory features, as well as discordant cytokine signaling. The addition of JAKi induced a more modest and limited increase in cytokine signaling [31]. Simultaneously, it rescued the phenotype of exhausted CD8 + T cells, as observed in mouse models of colon cancer, T cell lymphoma, lung cancer, and pancreatic cancer [31, 35, 39, 40]. Collectively, our findings highlight the potential of JAK inhibitors as valuable components in combination with ICI therapies to overcome ICI resistance, offering new strategies to enhance anti-tumor responses without compromising crucial immune functions, and emphasizing the importance of understanding the complex interplay between immune signaling pathways in cancer treatment. The optimal timing and duration of JAKi and ICI administration, along with the identification of markers distinguishing responders to dual ICI alone from those requiring JAKi, warrant further investigation in future studies.

### Supplementary Information

Below is the link to the electronic supplementary material.

Supplementary file1 (PDF 5364 KB)

## Data Availability

RNA sequence is available at NCBI GEO site accession number GSE250238. https://www.ncbi.nlm.nih.gov/geo/query/acc.cgi?acc=GSE250238
