# Supplementary material for: Janus kinase inhibitor overcomes resistance to immune checkpoint inhibitor treatment in peritoneal dissemination of gastric cancer in C57BL/6 J mice
Source: Gastric Cancer. 2024 May 28;27(5):971–85. doi: 10.1007/s10120-024-01514-5 (PMC11335826; doi:10.1007/s10120-024-01514-5)
Supplement: Supplementary file 1 — Supplementary file1 (PDF 5364 KB) [file 10120_2024_1514_MOESM1_ESM.pdf]

Article title: Janus kinase inhibitor overcomes resistance to immune checkpoint inhibitor treatment in peritoneal dissemination of gastric cancer in C57BL/6 J mice.

Journal: *Gastric Cancer*

Authors: Wan-Ying Du, Hiroki Masuda, Koji Nagaoka, Tomohiko Yasuda, Komei Kuge, Yasuyuki Seto, Kazuhiro Kakimi & Sachiyo Nomura

Corresponding author: Sachiyo Nomura (sachiyo.nomura1012@gmail.com)

Supplementary Materials

- Graphical Abstract
- Methods
- Supplementary Figures 1-6
- Supplementary Tables 1-2

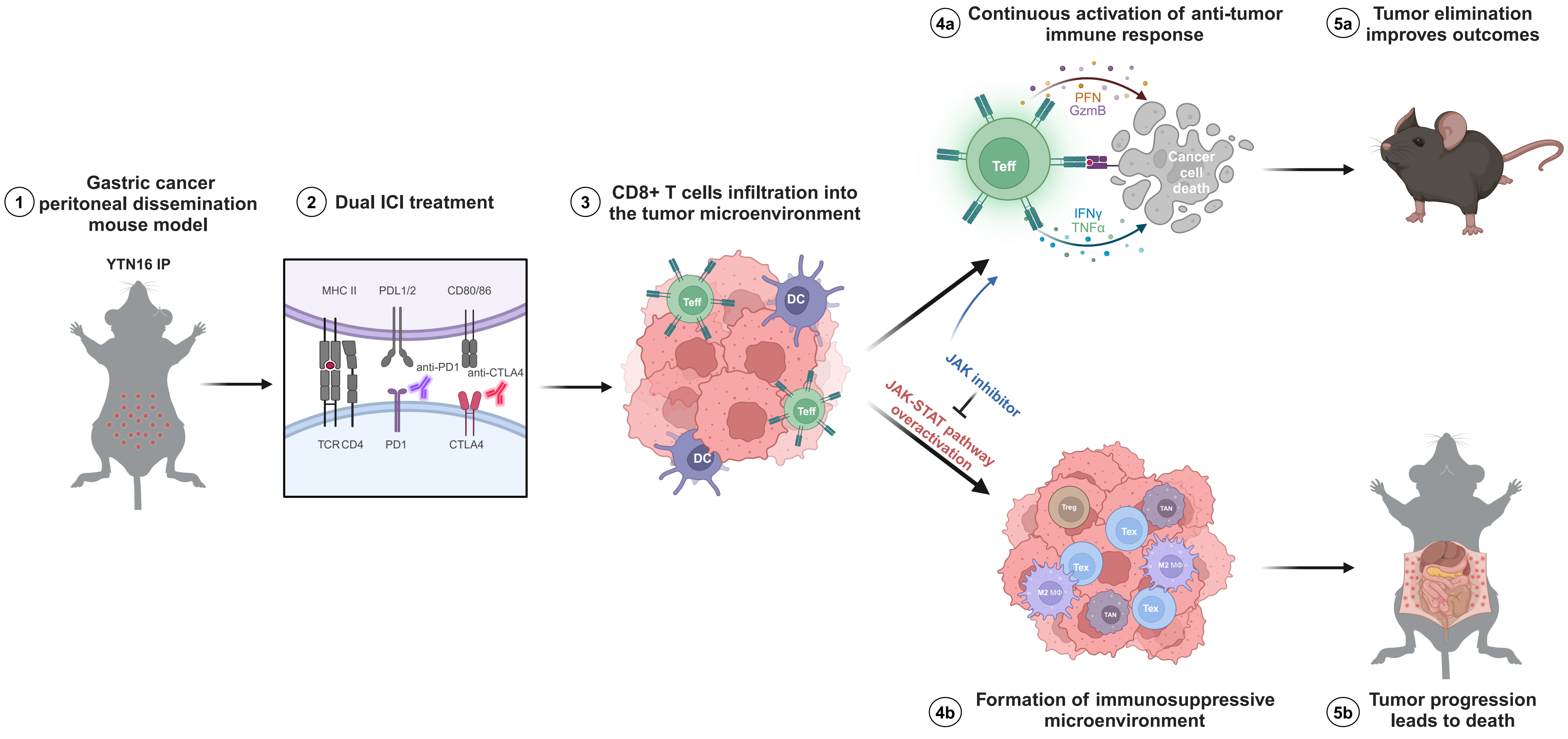

1 Gastric cancer peritoneal dissemination mouse model
YTN16 IP
2 Dual ICI treatment
MHC II
PDL1/2
CD80/86
anti-PD1
anti-CTLA4
TCR CD4
PD1
CTLA4
3 CD8+ T cells infiltration into the tumor microenvironment
Teff
DC
4a Continuous activation of anti-tumor immune response
PFN
GzmB
Teff
Cancer cell death
IFNγ
TNFα
5a Tumor elimination improves outcomes
JAK inhibitor
JAK-STAT pathway overactivation
Treg
TAN
Tex
M2 MΦ
4b Formation of immunosuppressive microenvironment
5b Tumor progression leads to death

Methods

Mouse model of peritoneal dissemination

YTN16 is a transplantable GC cell line in immunocompetent mice and is maintained in high-glucose Dulbecco's modified Eagle medium (DMEM, Sigma-Aldrich Japan, Japan) with supplements[13, 14].

The experiment was organized into 4 units, with female C57BL/6J mice (5-6-week-old, The Jackson Laboratory) were randomly divided into groups (supplementary Fig. 1A). Mice were kept in plastic cages with hardwood chips in an air-conditioned room with a 12 hour light-12 hour dark cycle and were given basal diet (CMF, Oriental Yeast Co., Tokyo, Japan) and water ad libitum. To establish peritoneal dissemination models, each mouse was intraperitoneally (IP) injected with 1×10^7 YTN16 cells in 500μl Hanks' Balanced Salt Solution (HBSS, Invitrogen Corporation) on day 0. All animal studies were conducted in accordance with the ARRIVE guideline and approved by The University of Tokyo Animal Care and Use Committee (P20-091), ensuring compliance with institutional guidelines.

Reagents and treatment protocols

For blocking immune checkpoint signaling, anti-PD1(10mg/kg) and/or CTLA-4(5mg/kg) mAbs, prepared in Phosphate Buffered Saline (PBS), were IP injected according to distinct timelines as illustrated in the respective figures (Figs. 1A, 2A, 5A, 6A), mice in the untreated control group were IP injected with PBS. For depletion of $CD4^+$ or $CD8^+$ T cells, anti-CD4 (10mg/kg) or CD8 (10mg/kg) mAbs were IP injected into the tumor bearing mice on days 4, 7 and 11. At 11, 21, and 35 days, mice were euthanized for subsequent analyses. For blocking of the JAK-STAT pathway, Ruxolitinib (LC Laboratories, Woburn, MA, USA) was dissolved in Dimethyl sulfoxide at 200mg/mL as a stock solution and further diluted in water containing 0.5% methylcellulose (w/v) and 0.1% Tween 80, then orally administered at a dose of 30mg/kg daily from day 8 post YTN16 cells implantation (Figs. 5A, 6A).

Flow cytometry

Briefly, peritoneal exudate cells were obtained by washing the abdominal cavity twice with pre-chilled PBS (5 mL each time), and peritoneally disseminated tumor

cells were derived from minced peritoneal tumor tissue. Both cell types were then incubated in RPMI-1640 (Nacalai Tesque, Japan) supplemented with 1% FBS, 10 mM HEPES, 0.2% collagenase (FUJIFILM Wako Pure Chemical Corporation, Osaka, Japan) and 2 KU/mL DNase I (Sigma-Aldrich, St. Louis, Missouri, USA) at 37℃ for 40 min. All material was passed through a 70 μm cell strainer to obtain single cell suspensions. After staining dead cells using the Zombie Yellow Fixable Viability Kit (BioLegend) and blocking of Fc receptors with anti-CD16/32 mAb (2.4G2, Bio X Cell), the cells were stained with the fluorescently conjugated mAbs for cell surface antigens. Stained cells were acquired on a CytoFLEX S flow cytometer (Beckman Coulter, Atlanta, Georgia, USA) and analyzed using FlowJo software V.10.6.2 (BD Biosciences).

Bulk RNA-sequence

Total RNA was extracted from the stripped peritoneum with or without disseminated nodules, using ISOGEN (Nippon Gene Co., Ltd., Japan) according to the manufacturer’s protocols. For analysis by RNA-sequence (RNA-seq), we ensured that RNA integrity was confirmed by Tape Station software, with the RNA integrity (RIN) score>8 (Agilent Technologies). Library preparation and sequencing (150 bp, paired-end reads) was performed as Illumina HiSeq standard protocols. The sequence reads were aligned to the mm10 reference genome using STAR V.2.5.2b. Mapped reads were counted by HTSeq V.0.6.1. Raw counts were normalized and differentially expressed genes (DEGs) were calculated using R software version 4.1.1 with DEseq2 and dplyr packages. For downstream analyses, we used STRING version 11.5 (https://www.string-db.org/), gene set enrichment analysis (GSEA, V.4.2.1) and TIMER2.0 platform (http://timer.cistrome.org/). The murine Microenvironment Cell Populations counter (mMCP-counter) algorithm was employed to estimate the relative abundance of different immune cell types within individual samples, enabling cross-sample comparisons. Additionally, we used the Cell-type Identification By Estimating Relative Subsets Of RNA Transcripts (CIBERSORT) algorithm to assess the relative proportions of various immune cell types within each individual sample. While CIBERSORT focuses on estimating immune cell proportions within individual samples,

mMCP-counter facilitates horizontal comparisons of immune cell composition across different samples. We used the OmicStudio tools (https://www.omicstudio.cn/tool) for visualization purposes, including the generation of heatmaps, volcano plots and bubble plots.

Immunohistochemistry staining and quantification

For immunohistochemistry, 4μm paraffin sections were deparaffinized, rehydrated, and antigen-retrieved using Immunosaver (Nishin EM, Tokyo, Japan) in a microwave. Sections were then treated with 3% H_2O_2/methanol to quench endogenous peroxidase activity and blocked with serum. Overnight incubation with primary antibodies (CD8α, Granzyme B, TBR2/Eomes, Foxp3, CD68, Ly6G, CD206, and p-STAT3; supplementary Table 1) was followed by visualization using VECTASTAIN Elite ABC HRP Kit and DAB Substrate Solution (VECTOR). Nuclei were stained with hematoxylin. The quantitative results of immunohistochemical staining for CD8, GZMB, Ly6G, Foxp3 were represented as the number of positive cells per tumor area. However, for CD68, CD206, and p-STAT3, counting was challenging due to the high number of stained cells, which could overlap. Therefore, we opted to express these results as the ratio of positive area to tumor area. Both counting methods were analyzed using ImageJ software across five high-power fields per sample.

Western Blotting

The samples were homogenized in T-PER (#78510, Thermo Fisher Scientific) with Protease and Phosphatase inhibitor (#78442, Thermo Fisher Scientific), and total protein concentration was determined using BCA protein assays (#23228, Thermo Fisher Scientific). Proteins (33μg per well) were loaded on 10% Mini-PROTEAN® TGX™ Precast Gels (#456-1035, BIO-RAD) and transblotted onto polyvinylidene fluoride membranes (Immobilon-P, Merck Millipore Ltd.). After blocking in 5% Skim Milk, membranes were incubated with primary antibodies (α/β-Tubulin, STAT3, p-STAT3; supplementary Table 1) and then with secondary antibodies. Detection was done using Trident femto-ECL Western Blotting Substrate (GTX14689, GeneTex) and imaged with MYECL Imager (Thermo Fisher Scientific). Band intensities were quantified using ImageJ software (NIH) after repeating the experiments 3 times.

Statistical analysis

For parametric data, the means among three or more groups were compared using one-way ANOVA followed by Tukey's multiple comparisons test. For non-parametric data or when the assumptions of normality and homogeneity of variances were not met, the Kruskal-Wallis test was used, followed by Dunn's multiple comparison test for post hoc analysis. All statistical analyses were performed using R software (version 4.1.1, R Foundation for Statistical Computing, Vienna, Austria) or Prism software version 9.0.0 (GraphPad Software, LLC, San Diego, CA, USA) at a significance level of $\alpha=0.05$. The data are presented as mean ± standard deviation (SD).

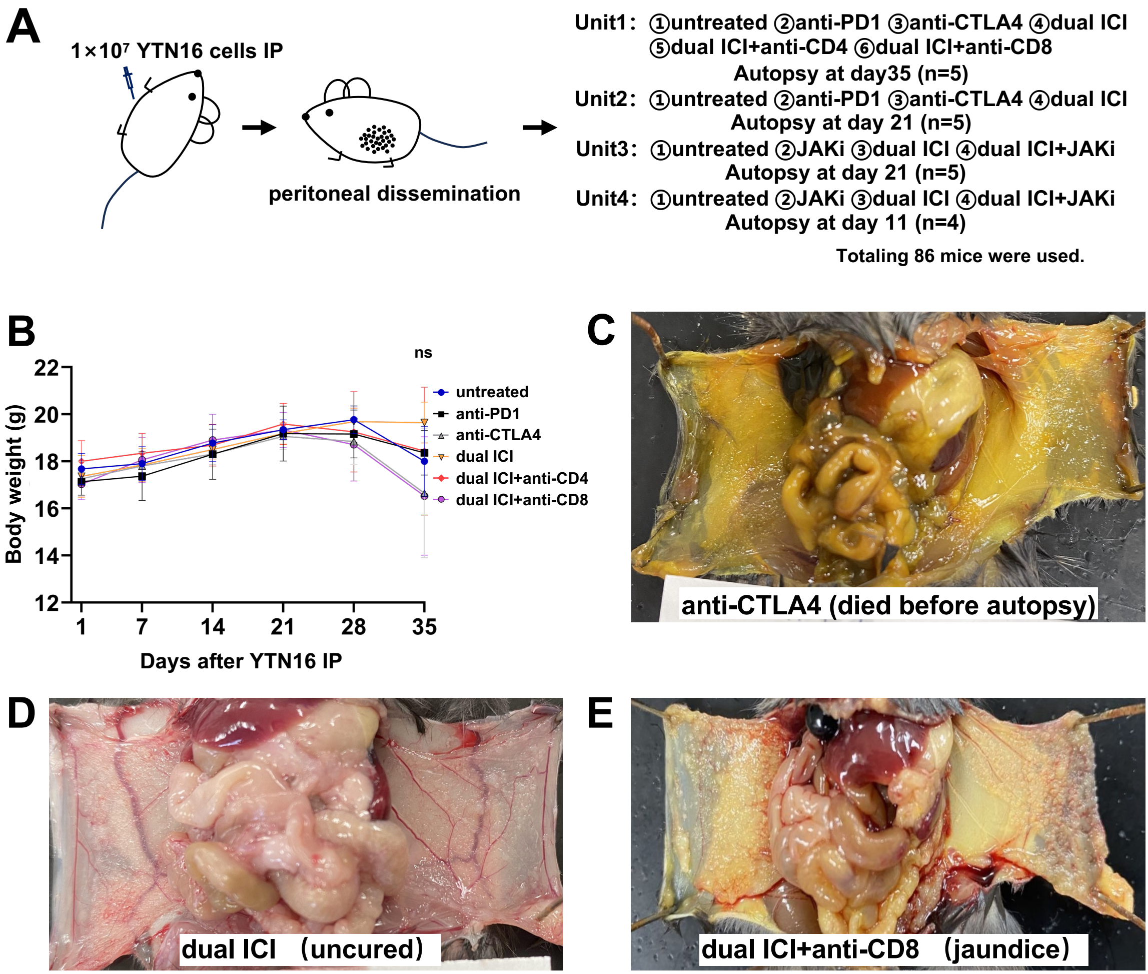

Supplementary Fig. 1 **A** Schematic diagram of mouse treatment grouping. **B** Changes in body weight of mice during treatment. **C** Macroscopic image of the mouse that died before autopsy in the anti-CTLA4 group. **D, E** Representative macroscopic images of peritoneal dissemination of mice in the dual ICI-uncured group and in the dual ICI+anti-CD8 group (jaundice). ns: not significant, one-way ANOVA with Tukey's multiple comparisons test

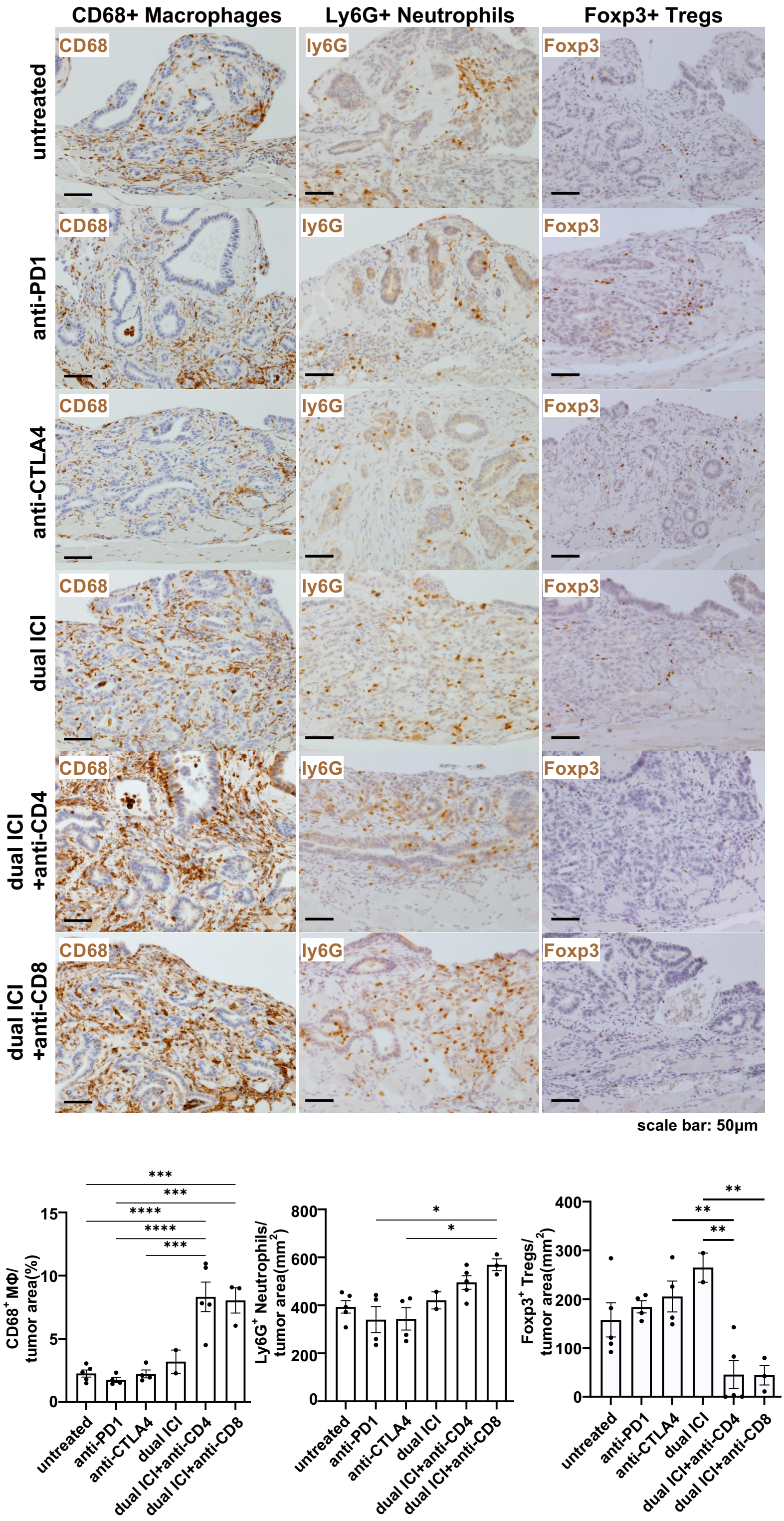

Supplementary Fig. 2 Representative images and quantitative analysis of IHC staining of CD68+ Macrophages, Ly6G+ Neutrophils and Foxp3+ Tregs on day 35. Scale bar: 50μm; **p*<0.05, ***p*<0.01, ****p*<0.001, *****p*<0.0001, one-way ANOVA with Tukey's multiple comparisons test

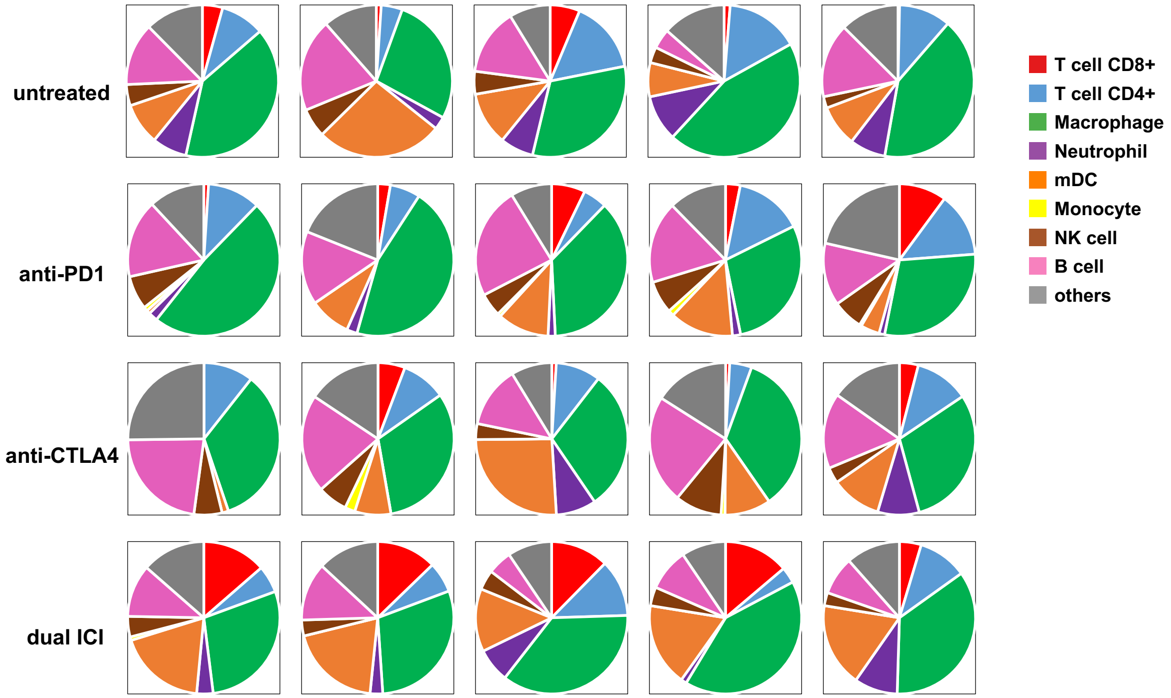

Supplementary Fig. 3 A pie chart format depicted the immune cell composition 3 weeks after treatment, as analyzed using CIBERSORT. The dual ICI treatment group exhibited an increased relative proportion of CD8+ T cells, whereas in the untreated group, neutrophils accounted for a higher proportion. CIBERSORT: Cell-type Identification By Estimating Relative Subsets Of RNA Transcripts (A specialized computational method for identifying and estimating the relative proportions of different cell types within complex tissues or cell mixtures)

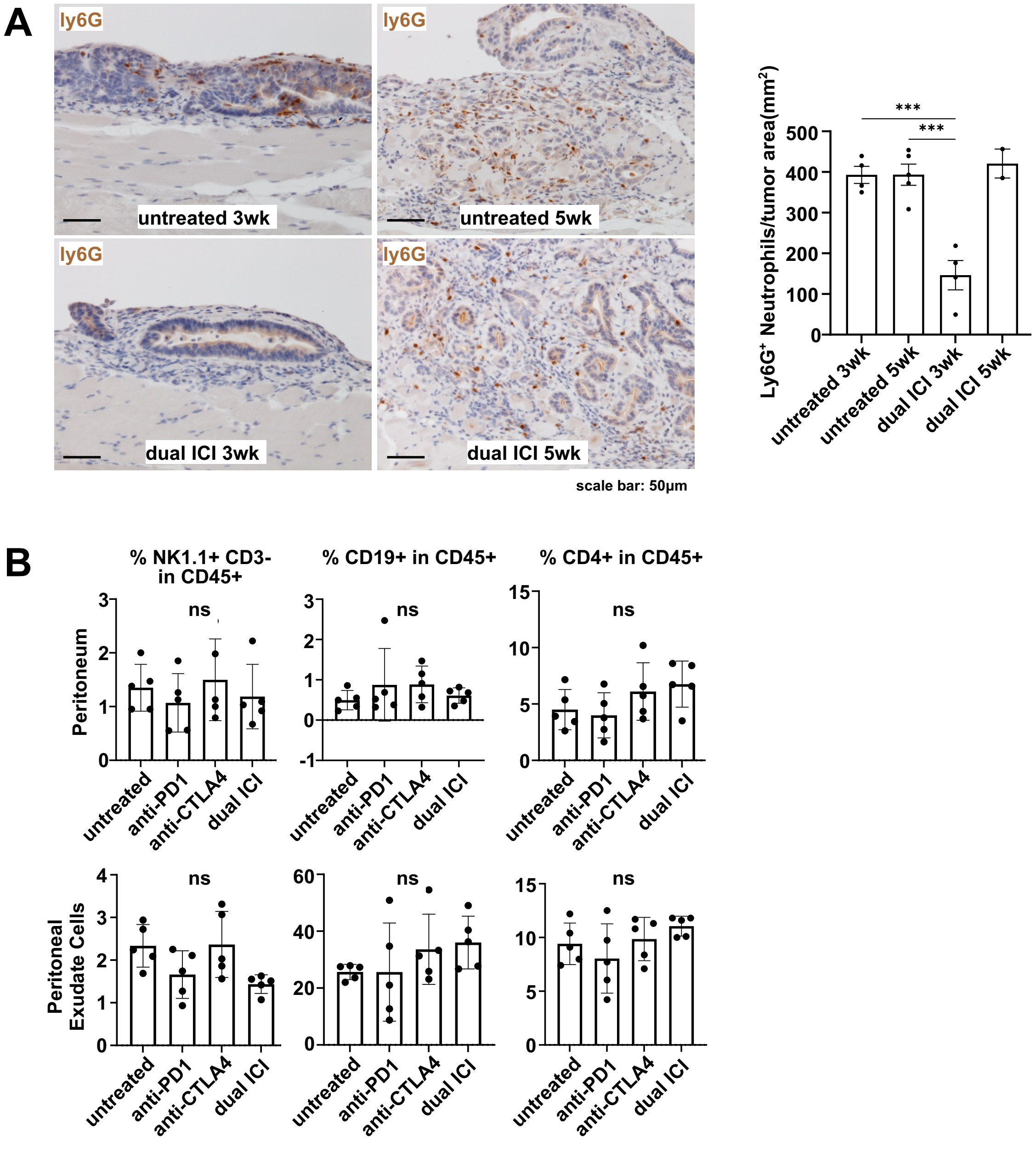

Supplementary Fig. 4 A Representative images of quantification of IHC staining of Ly6G+ Neutrophils at both early (3wk) and late (5wk) time points in the untreated and dual ICI treatment group. The Dual ICI treatment group exhibited significantly lower neutrophil infiltration at the early-stage. Scale bar: 50μm; ****p*<0.001, one-way ANOVA with Tukey's multiple comparisons test. **B** FACS was performed on peritoneal tumor and peritoneal exudate cells at the early time point (3wk). There were no significant differences observed in the quantity of NK cells, B cells, and CD4+ T cells among the groups. ns: not significant, Kruskal-Wallis test with Dunn’s multiple comparison

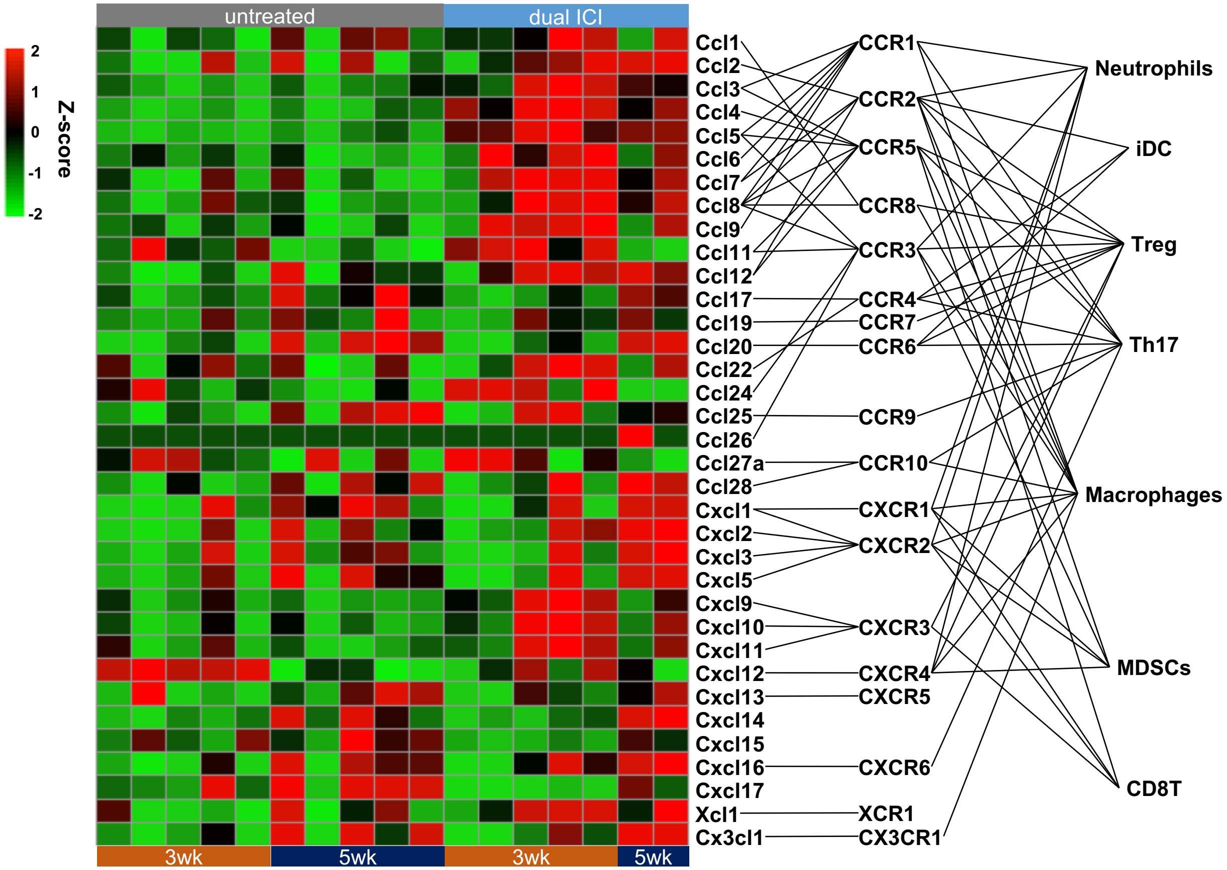

Supplementary Fig. 5 Heatmap of chemokine expression at both early (3wk) and late (5wk) time points in the untreated and dual ICI treatment group

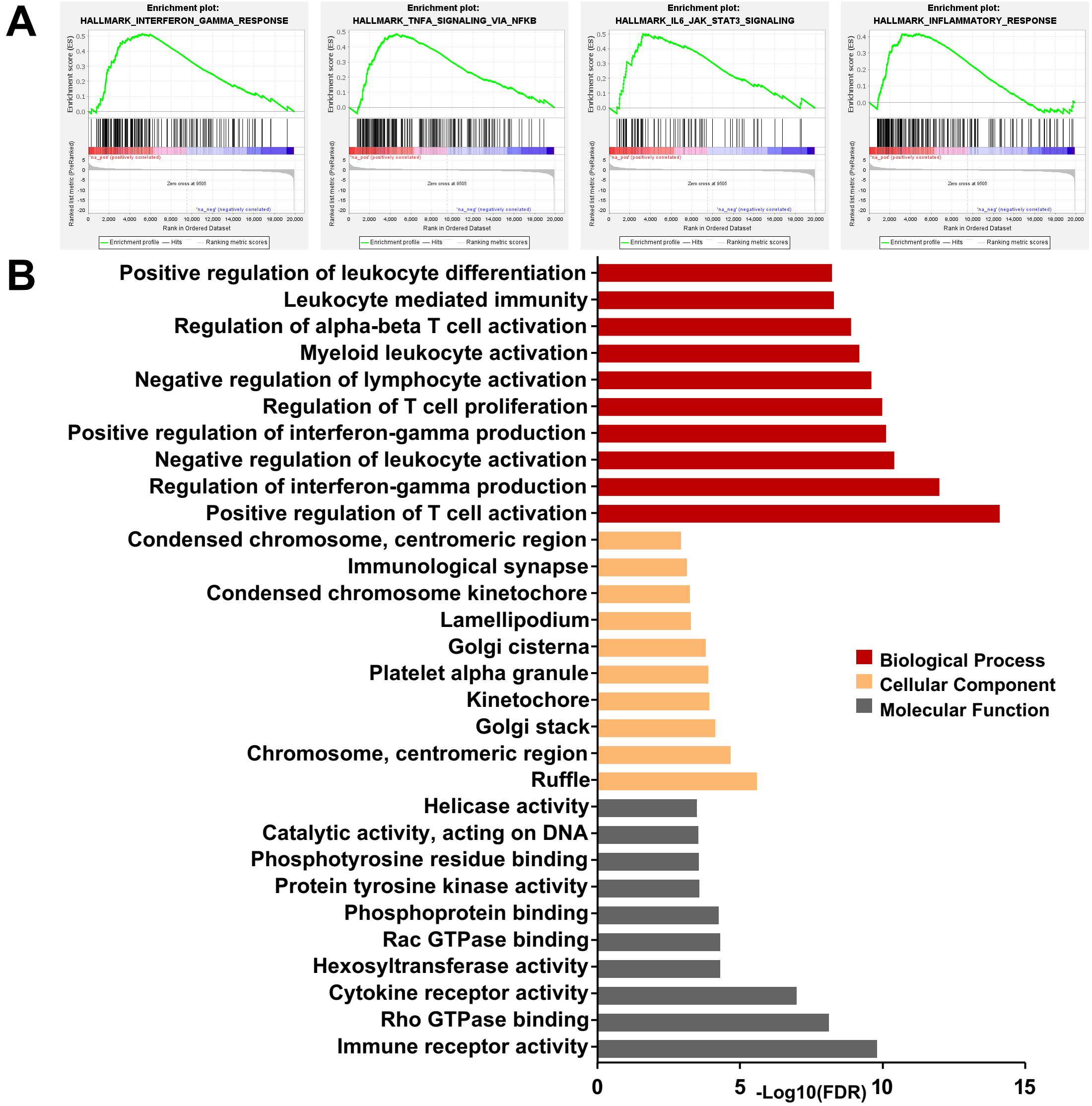

Supplementary Fig. 6 A The GSEA enrichment plot illustrated the enrichment scores of various gene sets across the ranked list of genes. The x-axis represents the genes, ordered based on their expression differences between the dual ICI-uncured group and the untreated group. The y-axis displays the enrichment score, indicating the degree of enrichment for each gene set at differentpoints along the ranked list. The clustering of black vertical lines indicated the positioning of pre-ranked genes within specific gene sets. The pronounced clustering observed in the red region signified the enrichment of these gene sets in the dual ICI-uncured group, highlighting their potential relevance to the observed resistance phenomenon. Conversely, a significant clustering in the blue region denoted enrichment in the untreated group. **B** A barplot displays the results of GO enrichment analysis for the categories of Biological Process, Cellular Component, and Molecular Function in dual ICI-uncured mice compared to untreated mice

Supplementary Table 1: List of Antibodies Used

Antibody	Brand	Clone Number	Catalog Number	Dilution or Dosage	Application
CD8α	Cell Signaling Technology (Danvers, Massachusetts, USA)	D4W2Z	#98941	1:200	Immunohistochemistry
Granzyme B (GZMB)	Cell Signaling Technology	E5V2L	#44153	1:200	Immunohistochemistry
TBR2/Eomes	Abcam(Cambridge, Massachusetts, USA)	EPR19012	#ab183991	1:800	Immunohistochemistry
Foxp3	Cell Signaling Technology	D6O8R	#12653	1:2000	Immunohistochemistry
CD68	Cell Signaling Technology	E3O7V	#97778	1:6000	Immunohistochemistry
Ly6G	Cell Signaling Technology	E6Z1T	#87048	1:400	Immunohistochemistry
CD206	Cell Signaling Technology	E6T5J	#24595	1:800	Immunohistochemistry
p-STAT3	Cell Signaling Technology	D3A7	#9145	1:600	Immunohistochemistry
α/β-Tubulin	Cell Signaling Technology	Polyclonal	#2148	1/2000	Western blotting
STAT3	Cell Signaling Technology	D1B2J	#30835	1/4000	Western blotting
p-STAT3	Cell Signaling Technology	D3A7	#9145	1/2000	Western blotting
PD-1	BioXcell (Lebanon, New Hampshire, USA)	RMP1-14	#BE0146	10mg/kg	Immune Checkpoint Inhibitor Treatment
CTLA-4	BioXcell	9H10	#BE0131	5mg/kg	Immune Checkpoint Inhibitor Treatment
CD4	BioXcell	GK1.5	#BP0003-1	10mg/kg	Cell Depletion
CD8α	BioXcell	53–6.7	#BE0004-1	10mg/kg	Cell Depletion

Supplementary Table 2:enrichment.NetworkNeighborAL

#term ID	term description	observed gene count	background gene count	strength	false discovery rate	matching proteins in your network (labels)
CL:16871	Mixed, incl. Apoptosis, and Toll/interleukin-1 receptor homology (TIR) domain	8	150	1.31	3.92E-05	Tnf,Il1r1,Il1b,Tnfrsf1a,Tnfsf13b,Casp3,Nfkb2,Bcl2
CL:16872	Mixed, incl. NF-kappa B signaling pathway, and TIR domain	6	96	1.38	0.00045	Tnf,Il1r1,Il1b,Tnfrsf1a,Tnfsf13b,Nfkb2
CL:25377	Dissolution of Fibrin Clot, and Extrinsic Pathway of Fibrin Clot Formation	4	19	1.9	0.00045	Plaur,F3,Serpine1,Proc
CL:16875	Mixed, incl. TNF receptor superfamily (TNFSF) members mediating non-canonical NF-kB pathway, and RIP-mediated NFkB activation via ZBP1	4	38	1.6	0.0036	Tnf,Tnfrsf1a,Tnfsf13b,Nfkb2
CL:16913	TNF receptor superfamily (TNFSF) members mediating non-canonical NF-kB pathway, and Tumor necrosis factor receptor 13C/17	3	19	1.78	0.0155	Tnf,Tnfrsf1a,Tnfsf13b
CL:11897	Mixed, incl. PI5P, PP2A and IER3 Regulate PI3K/AKT Signaling, and RET signaling	5	150	1.1	0.0239	Fgfr4,Flt3,Erbb2,Met,Fgfr2
CL:12045	FGFR2 ligand binding and activation, and betaKlotho-mediated ligand binding	3	26	1.64	0.0242	Fgfr4,Flt3,Fgfr2
CL:16572	JAK-STAT signaling pathway	4	81	1.27	0.0256	Kit,Il6,Il2rb,Csf3r
CL:11899	Mixed, incl. IGF1R signaling cascade, and vascular endothelial growth factor signaling pathway	4	99	1.19	0.0457	Fgfr4,Flt3,Met,Fgfr2
CL:16918	Tumor necrosis factor binding, and fractalkine production	2	5	2.18	0.0457	Tnf,Tnfrsf1a
CL:17031	Fever generation, and positive regulation of interleukin-1-mediated signaling pathway	2	5	2.18	0.0457	Il1r1,Il1b
CL:25381	Dissolution of Fibrin Clot	2	5	2.18	0.0457	Plaur,Serpine1
CL:8093	Mixed, incl. Grb2-Sos complex, and Protein kinase C, delta	2	5	2.18	0.0457	Src,Prkcd
